# Differences in midgut transcriptomes between resistant and susceptible strains of *Chilo suppressalis* to Cry1C toxin

**DOI:** 10.1186/s12864-020-07051-6

**Published:** 2020-09-14

**Authors:** Geng Chen, Yanhui Wang, Yanmin Liu, Fajun Chen, Lanzhi Han

**Affiliations:** 1 Department of Entomology, College of Plant Protection, Nanjing Agricultural University, Nanjing, 210095 China; 2 The State Key Laboratory for Biology of Plant Diseases and Insect Pests, Institute of Plant Protection, Chinese Academy of Agricultural Sciences, Beijing, 100193 China

**Keywords:** *Chilo suppressalis*, Transcriptome, Cry1C toxin, Difference, Resistance mechanism

## Abstract

**Background:**

*Chilo suppressalis* is a widespread rice pest that poses a major threat to food security in China. This pest can develop resistance to Cry toxins from *Bacillus thuringiensis* (*Bt*), threatening the sustainable use of insect-resistant transgenic *Bt* rice. However, the molecular basis for the resistance mechanisms of *C. suppressalis* to Cry1C toxin remains unknown. This study aimed to identify genes associated with the mechanism of Cry1C resistance in *C. suppressalis* by comparing the midgut transcriptomic responses of resistant and susceptible *C. suppressalis* strains to Cry1C toxin and to provide information for insect resistance management.

**Results:**

A *C. suppressalis* midgut transcriptome of 139,206 unigenes was de novo assembled from 373 million Illumina HiSeq and Roche 454 clean reads. Comparative analysis identified 5328 significantly differentially expressed unigenes (DEGs) between *C. suppressalis* Cry1C-resistant and -susceptible strains. DEGs encoding Bt Cry toxin receptors, aminopeptidase-P like protein, the ABC subfamily and alkaline phosphatase were downregulated, suggesting an association with *C. suppressalis* Cry1C resistance. Additionally, Cry1C resistance in *C. suppressalis* may be related to changes in the transcription levels of enzymes involved in hydrolysis, digestive, catalytic and detoxification processes.

**Conclusion:**

Our study identified genes potentially involved in Cry1C resistance in *C. suppressalis* by comparative transcriptome analysis. The assembled and annotated transcriptome data provide valuable genomic resources for further study of the molecular mechanisms of *C. suppressalis* resistance to Cry toxins.

## Background

The striped stem borer *Chilo suppressalis* Walker (Lepidoptera: Pyralidae) is a widespread rice pest that poses a major threat to food security throughout Asia, including China, India, Sri Lanka, Japan and Malaysia [1–3]. Excessive application of chemical pesticides has led to the rapid evolution of insect resistance [4]. Consequently, insect-resistant genetically modified rice expressing *Bacillus thuringiensis* (*Bt*) has been used as an alternative method to control rice stem borers. Indeed, insect-resistant transgenic rice lines expressing *Cry1A or Cry1C* insecticidal proteins have been effective in controlling *C. suppressalis* [5–10]. In particular, the *cry1C* rice line has robust prospects for commercial use in China because it shows high efficiency in suppressing the damage caused by the rice pest complex *C. suppressalis* and *Sesamia inferens*. Nonetheless, the future application of *Bt* rice is severely threatened by the evolution of insect resistance. Therefore, the development and implementation of precautionary insect resistance management (IRM) strategies are critical for the sustainable use of *Bt* rice.

Understanding the mode of Cry toxin action and the mechanisms that confer resistance of insects to Cry toxins not only enhances the control efficacy of pests but also facilitates the development of IRM strategies to delay insect resistance [11]. There are two hypotheses that have been proposed regarding the mode of Cry toxin action: the pore formation model and the signal transduction model [12–14]. Based on the pore formation model, protoxin is solubilized in the alkaline gut, digested by gut proteinase, and converted to the activated toxin, which then binds to the cadherin receptor located in the cell membrane of the insect midgut. The interaction between the activated toxin and cadherin receptor results in proteolytic cleavage and activated toxin oligomerization. These oligomers bind to glycosylphosphatidylinositol (GPI)-anchoring receptors, aminopeptidase N (APN) and alkaline phosphatase (ALP), among others; the oligomers are inserted into lipid raft membranes and form pores that affect the ionic balance across the cell membrane, causing cell death due to osmotic lysis [12, 13]. In contrast, the signal transduction model considers that the binding of the activated Bt toxin to specific receptors stimulates the G-protein coupled signalling pathway, leading to the activation of protein kinases [14]. Regardless of the model, conversion of the protoxin to the activated toxin by insect midgut proteases and the binding of the activated toxins to receptors on the surface of midgut cells are recognized as essential steps for toxicity [15]. Thus, decreased conversion of the protoxin to activated toxin and reduced toxin binding of Cry toxin to receptors are considered the most common mechanisms resulting in insect resistance to Cry toxins due to downregulation or mutation of the midgut proteinase and receptors [15, 16]. For example, decreased expression of the trypsin gene leading to incomplete activation of protoxin results in Cry1A resistance in *Helicoverpa armigera* and *Ostrinia nubilalis* [17–21]. Currently, more than four types of functional receptors, including cadherin [22], aminopeptidase N (APN) [23], alkaline phosphatase (ALP) [24], ATP-binding cassette (ABC) transporters [25, 26] and others, have been identified and verified in lepidopteran species. Additionally, reduced binding ability of Cry toxin to midgut receptors via a decrease in the activity and transcription of ALP or APN as well as mutations of APN, cadherin, and ABCC2 [14, 22, 25–28] lead to Cry1A resistance in *H. armigera*, *Heliothis virescens*, *Plutella xylostella* and *Bombyx. mori*.

In contrast to the Cry1A toxin, the Cry1C mode of action has not been described in detail, though it is also a pore-forming toxin. For lepidopteran species, APN and cadherin have been identified as Cry1C receptors in *Spodoptera littoralis* and *Spodoptera exigua* [29–31]. Moreover, reduced expression of SeAPN1 has been found in an *S. exigua* Cry1C-resistant strain [32]. Previous studies have also shown that Cry1C receptors exhibit low or no competition with Cry1A toxins [29, 33]. Moreover, Cry1C and Cry1A toxins specifically bind to distinct isoforms of APN in the brush border membrane of *Manduca sexta* [34]. In our previous study, we observed that Cry1A and Cry1C were able to recognize different binding proteins in the midgut of *C. suppressalis* [8]. Furthermore, we found that cadherin from the midgut of *C. suppressalis* played differential functional roles with regard to Cry1Ab and Cry1C intoxication [35]. Based on these findings, Cry1A and Cry1C toxins appear to have different modes of action in insects. Thus, understanding the molecular mechanism involved in Cry1C action is very important to better implement Cry1C alone or pyramided with Cry1A.

Relative receptors of Cry toxin, APN [36, 37], cadherin [35, 38] and ALP [39], have been identified in *C. suppressalis*, though some new receptors that may be involved in Cry toxin action have not been identified. In particular, the resistance mechanism of Cry toxin in *C. suppressalis* remains unknown. Furthermore, Cry1C has been shown to be particularly effective in controlling the rice pest complex *C. suppressalis* and *S. inferens*. Therefore, clarification of the molecular mechanism of Cry1C resistance in *C. suppressalis* appears to be particularly important.

RNA-sequencing (RNA-Seq) technology is widely applied for studying changes in gene expression in animals, plants and other organisms. In particular, Solexa/Illumina RNA-Seq and digital gene expression-based next-generation sequencing technology have been employed to identify Cry toxin resistance-related genes in *Ostrinia furnacalis*, *H. armigera* and *P. xylostella* [40–42].

In this study, we examined transcriptional differences between laboratory selected Cry1C-resistant (FZ1C) and -susceptible strains (FZS) of *C. suppressalis* by bioinformatics and RNA-Seq technologies. The differentially expressed transcripts were identified and further validated by quantitative real-time (qRT-PCR) analysis. The results of this study provide prospective genes and pathways contributing to resistance evolution and reveal the molecular mechanism of insect resistance to Cry toxin.

## Results

### Illumina sequencing analysis and de novo assembly

The number of raw RNA sequencing reads from susceptible (FZS) and resistant (FZ1C) strains of *C. suppressalis* ranged from 55,728,490 to 84,319,118, respectively (Table 1). The corresponding number of clean reads ranged from 54,571,128 to 83,168,312. After the clean reads were assembled by Trinity software, 139,206 unigenes of both susceptible and resistant strains of *C. suppressalis* with a mean length of 1290 bp and N50 value of 2343 bp (Table 2) were obtained. Among these, 86,912 unigenes were greater than 500 bp, accounting for 62.43% of the assembled unigenes.

**Table 1:** Summary of reads of the midgut transcriptome in Cry1C-resistant (FZ1C) and -susceptible (FZS) strains of *C. suppressalis*

Sample	Raw Reads	Clean Reads	Error (%)	Q20 (%)	Q30 (%)	GC Content (%)
FZS_1	60,059,702	58,557,796	0.03	97.26	92.6	48.05
FZS_2	60,328,774	58,254,422	0.03	97.25	92.63	47.72
FZS_3	57,731,528	56,530,878	0.03	97.21	92.53	47.03
FZ1C_1	55,728,490	54,571,128	0.03	96.82	91.68	46.9
FZ1C_2	84,319,118	83,168,312	0.03	97.4	92.83	46.85
FZ1C_3	63,356,582	62,354,194	0.03	95.88	89.68	47

**Table 2:** Summary of the length of assembled unigenes of the midgut transcriptome in Cry1C-resistant (FZ1C) and -susceptible (FZS) strains of *C. suppressalis*

Min length	Mean length	Median length	Max length	N50	N90	Total nucleotides
201	1290	675	28,518	2343	498	179,580,569

### Annotation of assembled unigenes

Annotation of the 139,206 unigene sequences from the midgut transcriptome of the susceptible (FZS) and resistant (FZ1C) strains of *C. suppressalis* was performed by Blast searches with a cut-off E-value of 1e-5 in the following databases: NR (NCBI non-redundant protein sequences), NT (NCBI non-redundant nucleotide sequences), PFAM (Protein family), KOG/COG (Clusters of Orthologous Groups of proteins), Swiss-Prot (a manually annotated and reviewed protein sequence database), KO (KEGG Orthology) and GO (Gene Ontology). According to the results, 40.18% of the unigenes were annotated in NR, 27.72% in NT, 31.41% in PFAM, 15.17% in KOG, 25.9% in Swiss-Prot, 14% in KO and 31.45% in GO (Additional file 1: Figure S1,). In Blastx homology searches with a cut-off E-value of 1e-5, 33.8, 18.0, 17.3, 1.9, 1.7, and 27.5% of the unigenes matched with *B. mori*, *P. xylostella*, *Danaus plexippus*, *Lasius. niger*, *Tribolium castaneum* and other insects, respectively (Additional file 2: Figure S2,). The two best matches were the lepidopteran species *B. mori* and *P. xylostella*.

### Gene ontology (GO) classification

**Fig. 1:**
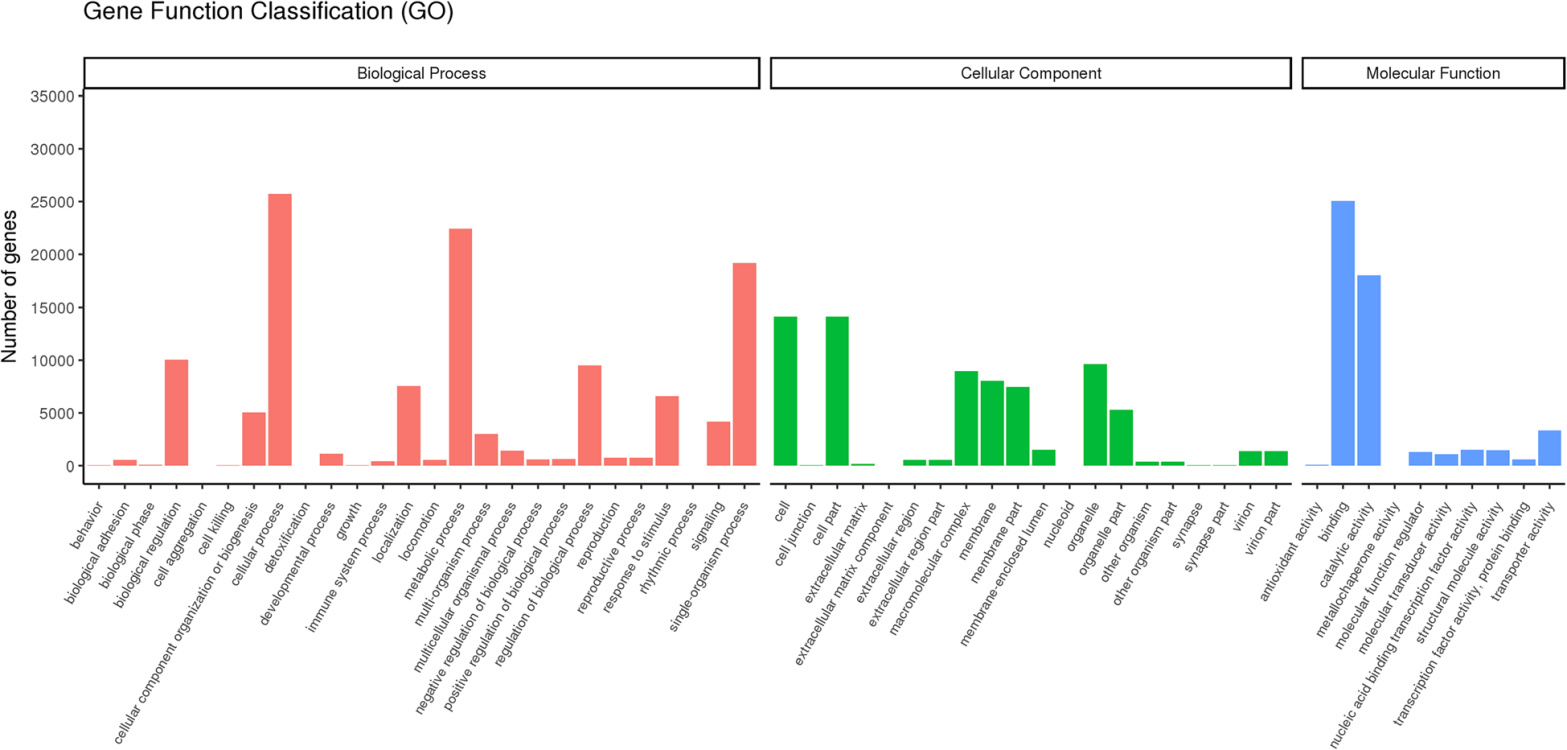
Histogram of Gene Ontology (GO) classification. The GO categories shown on the x-axis are grouped into three main ontologies: biological process, cellular component and molecular function. The y-axis indicates the number of unigenes in each category

GO annotation was conducted using Blast2GO software with a cut-off E-value 1e-6 to classify the unigenes into three GO classes, i.e., biological processes, cellular components and molecular functions. The unigenes were assigned into 56 functional groups (Fig. 1). Most were categorized as cellular process, metabolic process, single-organism process, biological regulation, cell, cell part, binding, and catalytic activity. Additionally, a high percentage of unigenes were assigned to the categories organelle, regulation of biological process, macromolecular complex, membrane, localization, membrane part, response to stimulus, organelle part, cellular component organization or biogenesis, signalling, transporter activity and multiorganism process (Fig. 1).

### KOG classification

**Fig. 2:**
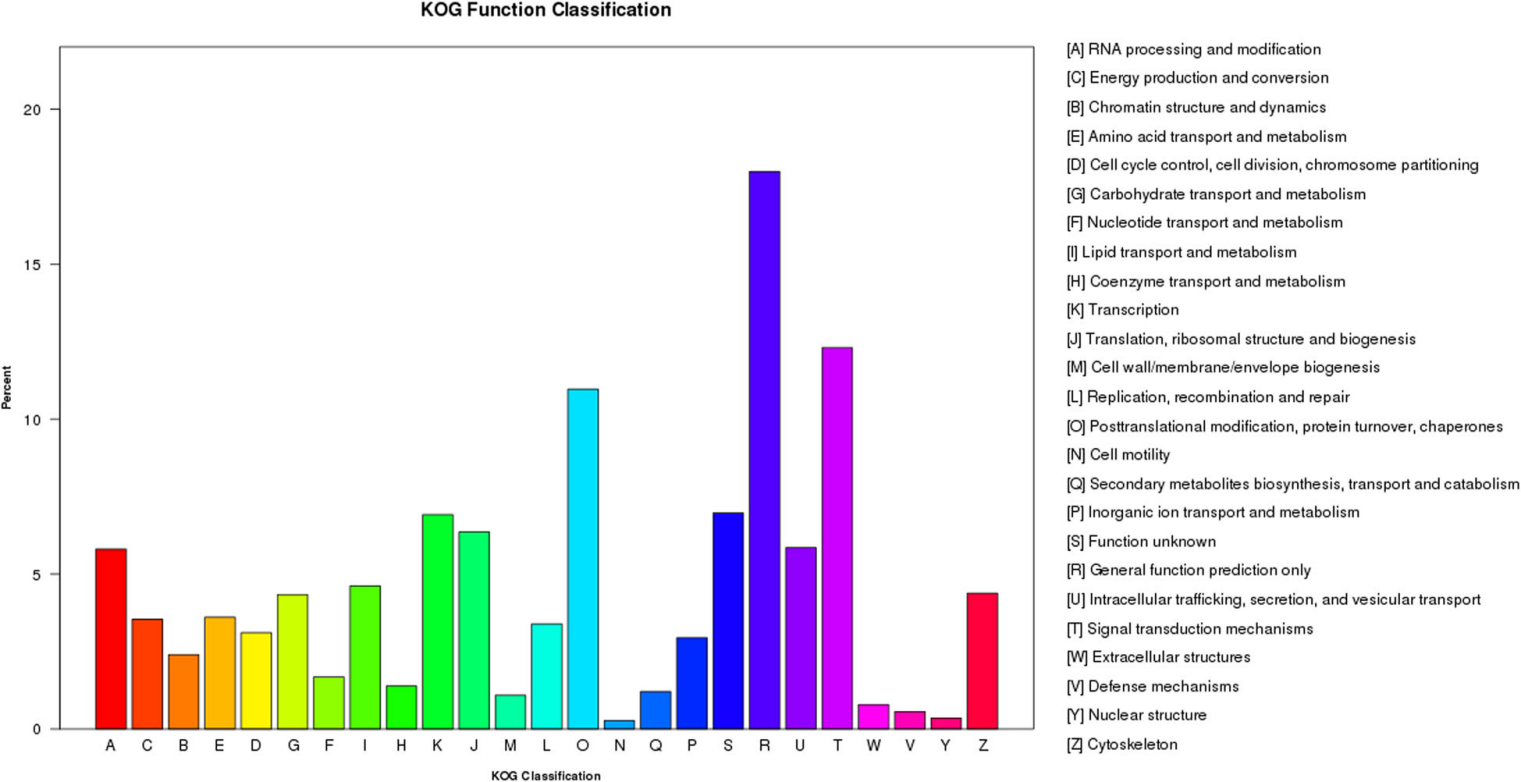
Histogram of clusters from the KOG classification. A total of 21,127 unigenes were assigned to 25 categories in the KOG classification. The right legend shows a description of the 25 functional categories

KOG is a database for the classification of orthologous genes. To further test the integrity and effectiveness of the annotation process, the unigenes annotated in the NR database were classified in KOG. In total, 21,127 unigenes were categorized into 25 KOG categories (A–W, Y and Z; Fig. 2), with “General function prediction only” accounting for the highest proportion among all the categories, followed by “Signal transduction mechanisms”, “Posttranslational modification, protein turnover, chaperones”, and “Transcription”. “Nuclear structure” and “Cell motility” occupied the lowest proportions of all categories (Fig. 2).

### Functional classification by KEGG

**Fig. 3:**
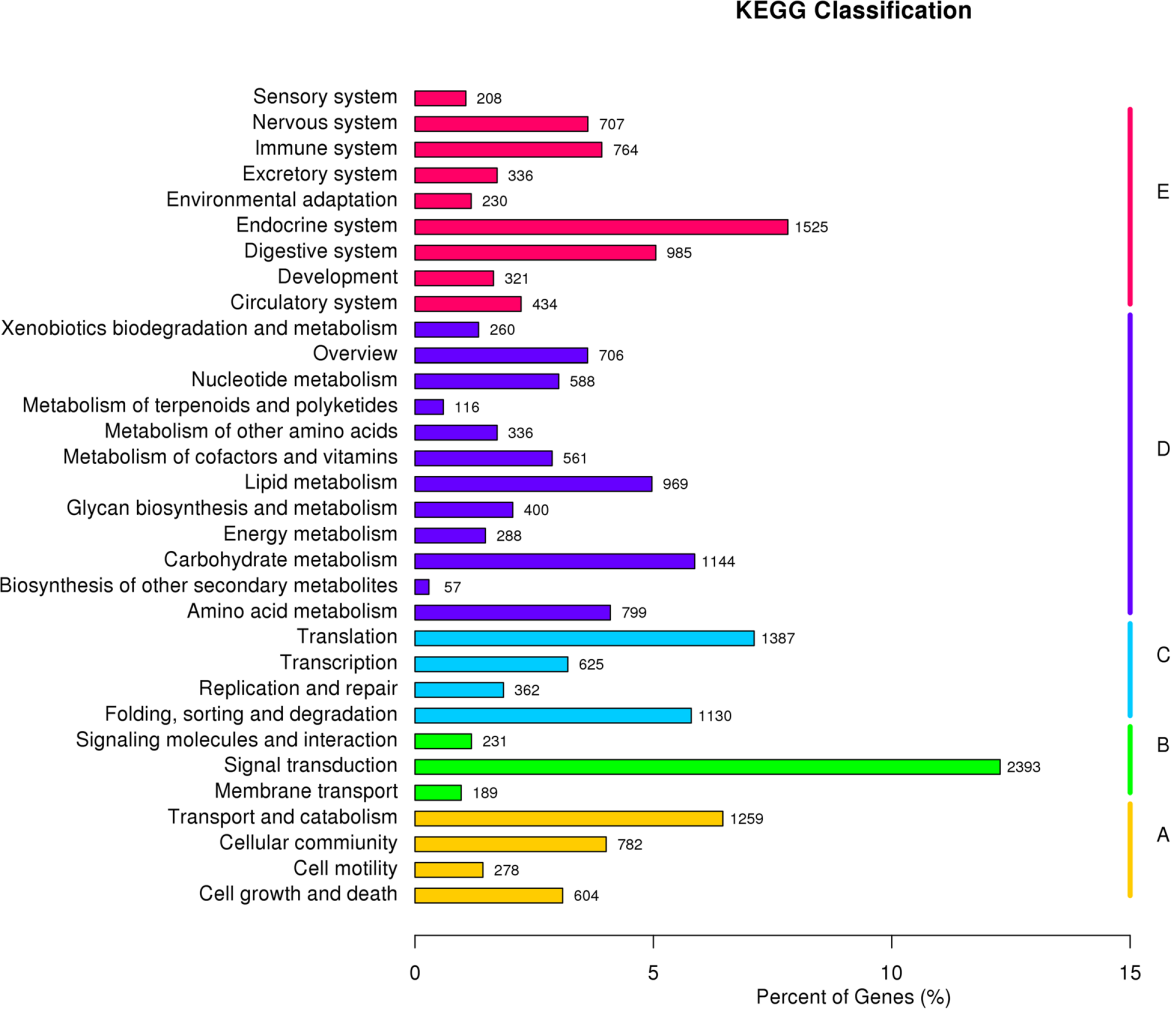
Histogram of KEGG functional classification. The Y-axis is the enrichment of KEGG terms, and the X-axis indicates the percentage of a specific category of genes in that main category. Genes were divided into five branches according to KEGG metabolic pathways: A –Cellular Processes; B –Environmental Information Processing; C –Genetic Information Processing; D –Metabolism; E –Organismal Systems

To gain further insight into the global network underlying the experimental treatments, KEGG pathway analysis was performed, whereby 19,498 annotated unigenes were mapped to a reference pathway. A total of 230 pathways were obtained, assigned into five major groups: cellular components (3400 unigenes), environmental information processing (5266 unigenes), genetic information processing (3920 unigenes), metabolism (8947 unigenes) and organismal systems (8783 unigenes) (Fig. 3).

### Differentially expressed unigenes (DEGs) among midgut transcripts

**Fig. 4:**
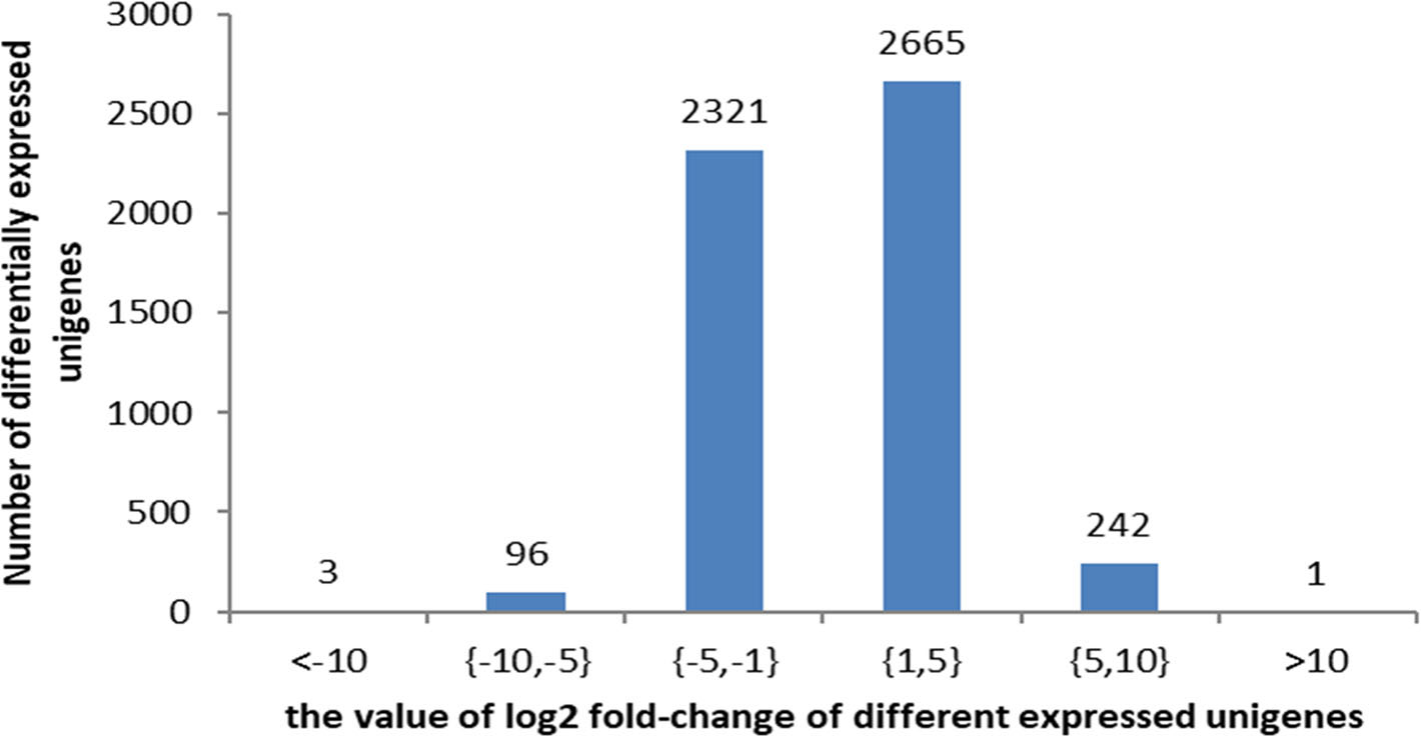
Changes in the distribution of differentially expressed unigenes between Cry1C-resistant (FZ1C) and Cry1C-susceptible (FZS) strains of *C. suppressalis*

Before the analysis of differentially expressed unigenes between the resistant and susceptible strains, the hierarchical clustering analysis on the midgut samples collected for transcriptome sequencing were conducted based on the filtered and normalized count matrix using the R package PVClust v.2.0. The hierarchical clustering analysis showed that three sample replications from C. suppressalis Cry1C-resistant or Cry1C-susceptible strain grouped in an individually respective cluster (Additional file 3: Figure S3). Moreover, the two clusters grouped were completely the same (Additional file 3: Figure S3), indicating no difference for the samples between Cry1C-resistant and Cry1C-susceptible strains. These suggest that the data from C. suppressalis midgut transcriptomes are repeatable and feasible. Then, the differentially expressed unigenes (DEGs) were analysed by comparing the midgut transcriptome of the FZ1C and FZS strains. The results revealed 5328 unigenes to be significantly differentially expressed in the midgut transcriptomes of the FZ1C and FZS strains (padj< 0.05, |log2fold-change| > 1). Among these, 2908 unigenes were upregulated and 2420 unigenes downregulated. Of all DEGs, 3 unigenes were downregulated and 1 upregulated more than 10 times, 96 unigenes were downregulated and 242 upregulated between 5 and 10 times, and 2321 unigenes were downregulated and 2665 upregulated between 1 and 5 times (Fig. 4).

The DEGs encoding potential Cry toxin receptors or those involved in the Cry toxin action pathway were further investigated (Table 3), including aminopeptidase-N, the ABC transporter family, alkaline phosphatase-like, trypsin, cadherin, V-type proton ATPase catalytic subunit A and heat shock proteins. Among them, nine unigenes encode APNs, but they were all upregulated in the FZ1C strain. Two unigenes annotated as an aminopeptidase P-like protein (APP-like) were both downregulated in the FZ1C strain; moreover, one of them was downregulated 10.741-fold compared with that in the FZS strain. Seventeen unigenes were associated with the ABC transporter family, 11 of which were upregulated and 6 downregulated in the FZ1C strain compared with the FZS strain. Ten unigenes encode ALPs: seven of them were downregulated and 3 of them upregulated in the FZ1C strain. Thirteen unigenes were annotated as trypsin or trypsin/chymotrypsinogen-like protein, with 12 of them being over-transcribed and 1 under-transcribed in the FZ1C strain. Three unigenes encode cadherin. One of them was downregulated, and 2 of them were upregulated. Two unigenes encoding a V-type proton ATPase catalytic subunit A were both upregulated. Four unigenes associated with heat shock proteins were all upregulated. The heatmap of DEGs encoding potential Cry toxin receptors or those involved in pathways of the mode of Cry toxin action are displayed (Additional file 4: Figure S4).

**Table 3:** The differential expression of candidate resistance genes between Cry1C-resistant (FZ1C) and -susceptible (FZS) strains of *C. suppressalis*

Gene_id	Log_**2**_ FoldChange	Padj	NR ID	NR Description
Cluster-21,897.55689	1.6449	7.39E-37	ABC69855.3	aminopeptidase N1 [*C. suppressalis*]
Cluster-21,897.55577	1.9015	6.17E-28	ABC69855.3	Aminopeptidase N1 [*C. suppressalis*]
Cluster-21,897.54666	1.33	7.80E-10	AFU51581.1	aminopeptidase N5 [*C. suppressalis*]
Cluster-21,897.55566	1.3123	3.64E-09	ABC69855.3	aminopeptidase N1 [*C. suppressalis*]
Cluster-21,897.57790	1.075	4.26E-06	AGG36452.1	aminopeptidase N2 [*C. suppressalis*]
Cluster-21,897.56678	2.1155	0.000903	AFU51581.1	aminopeptidase N5 [*C. suppressalis*]
Cluster-21,897.57101	1.4645	0.007899	AFU51581.1	aminopeptidase N5 [*C. suppressalis*]
Cluster-21,897.49282	1.0386	0.024454	ABC69855.3	aminopeptidase N1 [*C. suppressalis*]
Cluster-21,897.54390	1.2556	0.045792	ABC69855.3	aminopeptidase N1 [*C. suppressalis*]
Cluster-21,897.41443	−10.741	1.19E-62	ADZ74177.1	aminopeptidase P-like protein [*Ostrinianubilalis*]
Cluster-21,897.43493	−1.6657	0.011799	ADZ74177.1	aminopeptidase P-like protein [*O. nubilalis*]
Cluster-21,897.37634	1.7727	3.99E-07	AKC34899.1	ABCC1-like protein [*Spodopteralitura*]
Cluster-21,897.40893	3.4584	9.75E-25	XP_004926984.1	PREDICTED: ATP-binding cassette sub-family D member 2 [*Bombyx mori*]
Cluster-21,897.60875	1.6104	2.26E-13	XP_012546914.1	PREDICTED: ATP-binding cassette sub-family A member 1-like [*B. mori*]
Cluster-21,897.77353	1.8368	5.92E-11	XP_004926984.1	PREDICTED: ATP-binding cassette sub-family D member 2 [*B. mori*]
Cluster-21,897.6336	5.6608	1.58E-10	XP_012271259.1	PREDICTED: ATP-binding cassette sub-family G member 1-like [*Orussusabietinus*]
Cluster-21,897.52254	1.3426	0.000781	XP_012546914.1	PREDICTED: ATP-binding cassette sub-family A member 1-like [*B. mori*]
Cluster-25,303.0	3.0558	0.011063	XP_012240437.1	PREDICTED: ATP-binding cassette sub-family B member 7, mitochondrial isoform X3 [*Bombusimpatiens*]
Cluster-21,897.47836	1.7831	0.015241	ADV76536.1	ATP-binding cassette sub-family B member 1 [*Trichoplusiani*]
Cluster-21,897.94254	2.5774	0.021312	XP_007891965.1	PREDICTED: ATP-binding cassette sub-family B member 7, mitochondrial [*Callorhinchusmilii*]
Cluster-21,897.2225	2.8473	0.027446	XP_011261806.1	PREDICTED: ATP-binding cassette sub-family B member 7, mitochondrial isoform X1 [*Camponotusfloridanus*]
Cluster-21,897.39237	2.0934	0.032647	XP_012556248.1	PREDICTED: ATP-binding cassette sub-family G member 4-like [*Hydra vulgaris*]
Cluster-21,897.52385	−1.681	2.98E-10	AJD79133.1	ABCC3 [*C. suppressalis*]
Cluster-21,897.87849	−1.7765	0.012662	AJD79133.1	ABCC3 [*C. suppressalis*]
Cluster-21,897.77904	−1.4987	0.01506	AJD79133.1	ABCC3 [*C. suppressalis*]
Cluster-21,897.854	−3.5663	0.0019	AKC96179.1	ABC transporter subfamily C protein [*Plutellaxylostella*]
Cluster-21,897.54886	−1.3094	0.007259	XP_012551415.1	PREDICTED: ATP-binding cassette sub-family F member 2 isoform X1 [*B. mori*]
Cluster-21,897.58674	−1.2038	0.034899	EHJ74419.1	ATP-binding cassette sub-family C member 4 [*Danausplexippus*]
Cluster-21,897.109470	−6.9167	4.46E-18	XP_004923692.2	PREDICTED: membrane-bound alkaline phosphatase-like [*B. mori*]
Cluster-21,897.58470	−2.2938	0.016426	XP_004926653.1	PREDICTED: membrane-bound alkaline phosphatase-like [*B. mori*]]
Cluster-21,897.75922	−3.1934	3.39E-05	AHF20243.1	membrane-bound alkaline phosphatase [*P. xylostella*]
Cluster-21,897.56465	−1.7759	8.36E-05	AGG36455.1	alkaline phosphatase 3 [*C. suppressalis*]
Cluster-21,897.58789	−2.549	5.46E-14	AGG36453.1	alkaline phosphatase 1 [*C. suppressalis*]
Cluster-21,897.40486	−2.1239	0.010296	AGG36453.1	alkaline phosphatase 1 [*C. suppressalis*]
Cluster-21,897.24953	−2.6531	0.043486	AGG36453.1	alkaline phosphatase 1 [*C. suppressalis*]
Cluster-21,897.35257	6.9134	1.66E-16	AGG36453.1	alkaline phosphatase 1 [*C. suppressalis*]
Cluster-21,897.56483	1.5945	7.02E-05	AFI81421.1	alkaline phosphatase 1 [*Diatraeasaccharalis*]
Cluster-21,897.58285	1.5901	0.009408	AGG36455.1	alkaline phosphatase 3 [*C. suppressalis*]
Cluster-21,897.57556	3.1885	4.07E-18	AFK64821.1	trypsin-like proteinase [*C. suppressalis*]
Cluster-21,897.56622	1.3654	8.59E-10	AAC36150.1	chymotrypsinogen-like protein [*Plodiainterpunctella*]
Cluster-21,897.56271	1.5268	2.57E-07	AAC02220.1	putative trypsin-like protein [*Scirpophagaincertulas*]
Cluster-21,897.55915	1.0453	4.69E-07	AFK64828.1	trypsin-like proteinase [*C. suppressalis*]
Cluster-21,897.56673	1.5238	6.31E-05	AFK64821.1	trypsin-like proteinase [*C. suppressalis*]
Cluster-21,897.55582	1.1248	8.67E-05	AAR98920.2	trypsin-like proteinase T2a precursor [*O.nubilalis*]
Cluster-21,897.35243	1.7242	0.000309	AAC02220.1	putative trypsin-like protein [*S. incertulas*]
Cluster-21,897.55247	1.4464	0.003929	AFK64824.1	trypsin-like proteinase [*C. suppressalis*]
Cluster-21,897.54394	1.2619	0.00564	AFK64824.1	trypsin-like proteinase [*C. suppressalis*]
Cluster-21,897.55799	1.2883	0.017361	AAC02220.1	putative trypsin-like protein [*S. incertulas*]
Cluster-21,897.58977	1.3923	0.026063	AAR98920.2	trypsin-like proteinase T2a precursor [*O. nubilalis*]
Cluster-21,897.54072	1.1272	0.044035	AFK64828.1	trypsin-like proteinase [*C. suppressalis*]
Cluster-21,897.40330	−1.4309	0.029499	ACB54938.1	trypsin [*Helicoverpaarmigera*]
Cluster-21,897.53425	2.2012	6.83E-05	ACY69027.1	mutant cadherin [*H. armigera*]
Cluster-21,897.66616	2.8159	0.031303	ACY69027.1	mutant cadherin [*H. armigera*]
Cluster-21,897.72833	− 2.4799	0.030472	ACY69027.1	mutant cadherin [*H. armigera*]
Cluster-21,897.55855	1.5892	2.41E-05	EHJ78293.1	V-type proton ATPase catalytic subunit A [*D. plexippus*]
Cluster-13,786.0	2.6297	0.035587	XP_976188.1	PREDICTED: V-type proton ATPase catalytic subunit A [*Triboliumcastaneum*]
Cluster-21,897.55072	1.1929	8.41E-21	NP_001266403.1	heat shock protein 90 beta [*B. mori*]
Cluster-21,897.68076	4.4643	7.65E-06	XP_004931165.1	PREDICTED: heat shock protein 67B2-like [*B. mori*]
Cluster-2235.0	4.1034	9.04E-05	EJY88528.1	Heat shock protein 90 [*Oxytrichatrifallax*]
Cluster-4673.0	3.0708	0.012694	AAM93752.1	heat shock protein 90, partial [*Cryptobiahelicis*]
Cluster-21,897.55634	1.4418	4.86E-10	ACI45417.1	chymotrypsin-like serine proteinase C3 [*O. nubilalis*]
Cluster-21,897.55418	1.187	1.60E-09	ACI45417.1	chymotrypsin-like serine proteinase C3 [*O. nubilalis*]
Cluster-21,897.55499	1.1561	1.82E-09	ACI45417.1	chymotrypsin-like serine proteinase C3 [*O. nubilalis*]
Cluster-21,897.56181	1.3734	1.22E-08	ACR15986.2	serine protease 11 [*Mamestraconfigurata*]
Cluster-21,897.55981	1.2165	2.45E-08	ACR15986.2	serine protease 11 [*M. configurata*]
Cluster-21,897.54910	1.4828	5.86E-06	ACI45413.1	trypsin-like serine proteinase T21 [*O. nubilalis*]
Cluster-21,897.54375	1.6568	2.53E-05	ACI45417.1	chymotrypsin-like serine proteinase C3 [*O. nubilalis*]
Cluster-21,897.102495	3.2954	0.005237	AFQ01141.1	serine protease inhibitor 005 [*H. armigera*]
Cluster-21,897.85135	4.3327	5.91E-12	AEW46893.2	serine protease inhibitor 002 [*H. armigera*]
Cluster-21,897.83284	−2.6304	1.92E-10	AIR09773.1	chymotrypsin-like serine protease precursor [*Spodopterafrugiperda*]
Cluster-21,897.24583	−3.5303	8.09E-10	AGU27161.1	serine protease 13 [*Antheraeapernyi*]
Cluster-21,897.55169	−1.061	4.53E-09	AAX62026.1	chymotrypsin-like serine protease [*O. nubilalis*]
Cluster-21,897.48503	−1.9352	7.19E-09	AFM77775.1	chymotrypsin-like serine protease 16 [*O. nubilalis*]
Cluster-21,897.48502	−1.499	2.11E-08	AFM77775.1	chymotrypsin-like serine protease 16 [*O. nubilalis*]
Cluster-21,897.62833	−2.0668	3.22E-06	AFM77775.1	chymotrypsin-like serine protease 16 [*O. nubilalis*]
Cluster-21,897.107082	−2.4889	0.013375	XP_012545493.1	PREDICTED: clip domain serine protease 4 isoform X1 [*B. mori*]
Cluster-21,897.104728	−2.9256	0.020799	ACD44927.1	serine protease [*B. mandarina*]
Cluster-24,270.0	−1.6631	0.027819	EHJ71121.1	serine protease P54 [*D. plexippus*]
Cluster-21,897.52100	−2.2617	6.60E-23	EHJ69197.1	putative serine protease inhibitor dipetalogastin precursor [*D. plexippus*]
Cluster-21,897.53494	−2.3869	3.90E-21	EHJ69197.1	putative serine protease inhibitor dipetalogastin precursor [*D. plexippus*]
Cluster-21,897.56879	−1.901	2.31E-18	EHJ63159.1	serine protease inhibitor 28 [*D. plexippus*]
Cluster-21,897.55723	−2.1363	2.52E-18	EHJ69197.1	putative serine protease inhibitor dipetalogastin precursor [*D. plexippus*]
Cluster-21,897.49312	−2.7939	3.65E-11	AEW46890.2	serine protease inhibitor 004, partial [*C. uppressalis*]
Cluster-21,897.49308	−2.7708	1.94E-10	AEW46890.2	serine protease inhibitor 004, partial [*C. uppressalis*]
Cluster-21,897.65582	−1.7348	7.84E-10	EHJ63159.1	serine protease inhibitor 28 [*D. plexippus*]
Cluster-21,897.65709	−3.4051	8.00E-10	NP_001139719.1	serine protease inhibitor 28 [*B. mori*]
Cluster-21,897.61734	−2.0756	3.20E-09	EHJ69197.1	putative serine protease inhibitor dipetalogastin precursor [*D. plexippus*]
Cluster-21,897.49522	−2.8872	0.000267	EHJ63159.1	serine protease inhibitor 28 [*D. plexippus*]
Cluster-21,897.73556	−2.6745	0.000291	AEW46890.2	serine protease inhibitor 004, partial [*C. suppressalis*]
Cluster-21,897.97023	−3.8594	0.000413	AFQ01141.1	serine protease inhibitor 005 [*C. suppressalis*]
Cluster-21,897.43046	−2.2187	0.002689	AEW46890.2	serine protease inhibitor 004, partial [*C. uppressalis*]
Cluster-21,897.49311	−2.8937	0.006279	AEW46890.2	serine protease inhibitor 004, partial [*C. uppressalis*]
Cluster-21,897.54219	−1.1075	0.006562	AEW46891.2	serine protease inhibitor 012 [*C. uppressalis*]
Cluster-21,897.42809	−3.0869	0.011017	AEW46893.2	serine protease inhibitor 002 [*C. uppressalis*]
Cluster-21,897.49615	−1.6659	0.011354	EHJ63159.1	serine protease inhibitor 28 [*D. plexippus*]
Cluster-23,305.0	−1.9878	0.041682	EHJ72371.1	putative pacifastin-related serine protease inhibitor [*D. plexippus*]
Cluster-21,897.71957	−1.9897	0.017717	AEW46890.2	serine protease inhibitor 004, partial [*C. uppressalis*]
Cluster-21,897.49313	−2.862	0.002877	XP_974161.2	PREDICTED: antichymotrypsin-2-like [*T. castaneum*]
Cluster-21,897.20361	−2.4984	1.82E-05	BAM18141.1	cytochrome P450 6a2 [*Papilio xuthus*]
Cluster-21,897.108883	−3.2084	0.0087607	AJN91180.1	cytochrome P450 monooxygenase CYP324A19 [Cnaphalocrocis medinalis]
Cluster-21,897.3843	5.3522	3.77E-09	AJN91157.1	cytochrome P450 monooxygenase CYP301A1 [Cnaphalocrocis medinalis]
Cluster-21,897.18070	3.0411	0.016058	AJN91157.1	cytochrome P450 monooxygenase CYP301A1 [Cnaphalocrocis medinalis]
Cluster-21,897.66501	−3.9796	0.00022976	AEL33702.1	carboxylesterase CXE29 [Spodoptera littoralis]
Cluster-21,897.65624	−3.1944	0.0035667	ADD97157.1	carboxylesterase [Helicoverpa armigera]
Cluster-21,897.36694	−1.5948	0.019801	ADA83700.1	carboxylesterase [Helicoverpa armigera]
Cluster-21,897.53957	1.2891	1.26E-44	AIY69075.1	carboxylesterase, partial [Chilo suppressalis]
Cluster-21,897.54856	1.1913	2.42E-08	NP_001121786.1	alpha-esterase 3 precursor [Bombyx mori]
Cluster-21,897.58344	1.5009	7.64E-07	AIY69075.1	carboxylesterase, partial [Chilo suppressalis]
Cluster-21,897.37880	4.8718	8.14E-07	AJN91204.1	carboxylesterase [Cnaphalocrocis medinalis]
Cluster-21,897.51529	2.4267	2.31E-06	AIY69074.1	carboxylesterase [Chilo suppressalis]
Cluster-21,897.86249	4.6063	4.85E-06	AJN91204.1	carboxylesterase [Cnaphalocrocis medinalis]
Cluster-21,897.51354	2.843	7.93E-05	NP_001121786.1	alpha-esterase 3 precursor [Bombyx mori]
Cluster-21,897.56317	2.0269	0.0048069	AIY69040.1	carboxylesterase [Chilo suppressalis]
Cluster-21,897.58734	1.394	0.0053925	AIY69040.1	carboxylesterase [Chilo suppressalis]
Cluster-21,897.55465	2.0319	0.0055622	AIY69075.1	carboxylesterase, partial [Chilo suppressalis]
Cluster-21,897.44386	1.1115	0.014022	XP_004923385.2	PREDICTED: LOW QUALITY PROTEIN: venom carboxylesterase-6-like [Bombyx mori]
Cluster-21,897.58907	1.1912	0.021256	NP_001121786.1	alpha-esterase 3 precursor [Bombyx mori]
Cluster-21,897.83149	−1.5141	1.05E-10	AII16887.1	microsomal glutathione S-transferase [Antheraea yamamai]
Cluster-21,897.94648	−4.2961	2.79E-05	AIH07599.1	glutathione S-transferase zeta 2 [Spodoptera litura]

In this study, we not only investigated DEGs encoding potential Cry toxin receptors but also examined DEGs encoding detoxification enzymes, such as cytochrome P450 (P450), carboxylesterase (CaE) and glutathione S-transferase (GST) (Table 3). There were 15 unigenes encoding CaE; 12 of them were upregulated, and 3 of them were downregulated. Four unigenes encoding P450 were upregulated, and the other 2 unigenes were downregulated. The 2 DEGs encoding GST were both downregulated. Moreover, some putative glutamate receptors targeting insecticides were identified.

Immune genes involved in haemolymph melanization, such as serpin protease and serpin protease inhibitor, were also examined (Table 3). The results showed 17 unigenes encoding serpin proteases. Seven of them were upregulated and 10 downregulated in the resistant strain compared with the susceptible strain. Twenty-two unigenes encode serpin protease inhibitors: two of them were upregulated and the other 20 downregulated in the Cry1C-resistant strain compared to the susceptible strain (Table 3). A heatmap of DEGs encoding serpin proteases and serpin protease inhibitors is exhibited (Additional file 4: Figure S4, F and G).

**Fig. 5:**
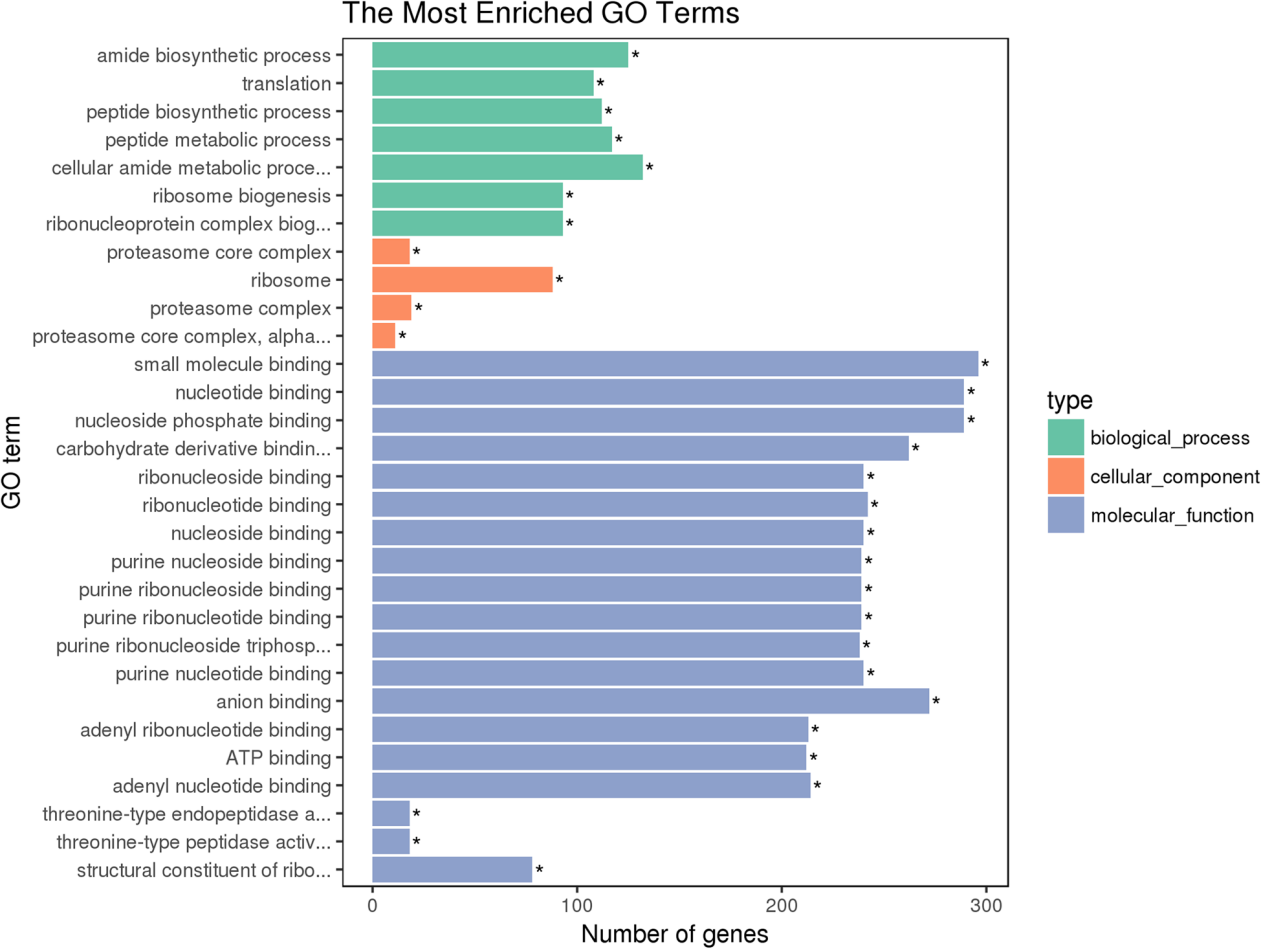
Gene Ontology (GO) classification of upregulated unigenes from the Cry1C-resistant strain of *C. suppressalis*

To further analyse the biological functions of the significant DEGs between the resistant (FZ1C) and susceptible (FZS) strains of *C. suppressalis*, GO functional and pathway enrichment analyses were carried out. The upregulated unigenes were annotated to 1727, 378 and 761 GO terms in the biological process, cellular component and molecular function categories, respectively. The top 30 GO terms (according to corrected *P*-values) are displayed in Fig. 5, including 7 GO terms for biological processes, 4 GO terms for cellular components and 19 GO terms for molecular functions. Among the 7 biological processes, the amide biosynthetic process (GO: 0043604, corrected *P*-value = 3.87e^− 07^) was the most enriched GO term. The cellular amide metabolic process (GO: 0043603, corrected *P*-value = 1.89e^− 06^) was the largest, with 132 upregulated unigenes. Among the 4 cellular component GO terms, the proteasome core complex term (GO: 0005839, corrected *P*-value = 1.61e^− 06^) was the most represented, and the ribosome (GO: 0005840, corrected *P*-value = 4.89e^− 06^) was the largest category, with 88 upregulated unigenes. For the significantly enriched GO terms in “molecular function”, small molecule binding (GO: 0036094, corrected *P*-value = 1.01e^− 10^) was the most enriched and largest category, with 296 upregulated unigenes.

**Fig. 6:**
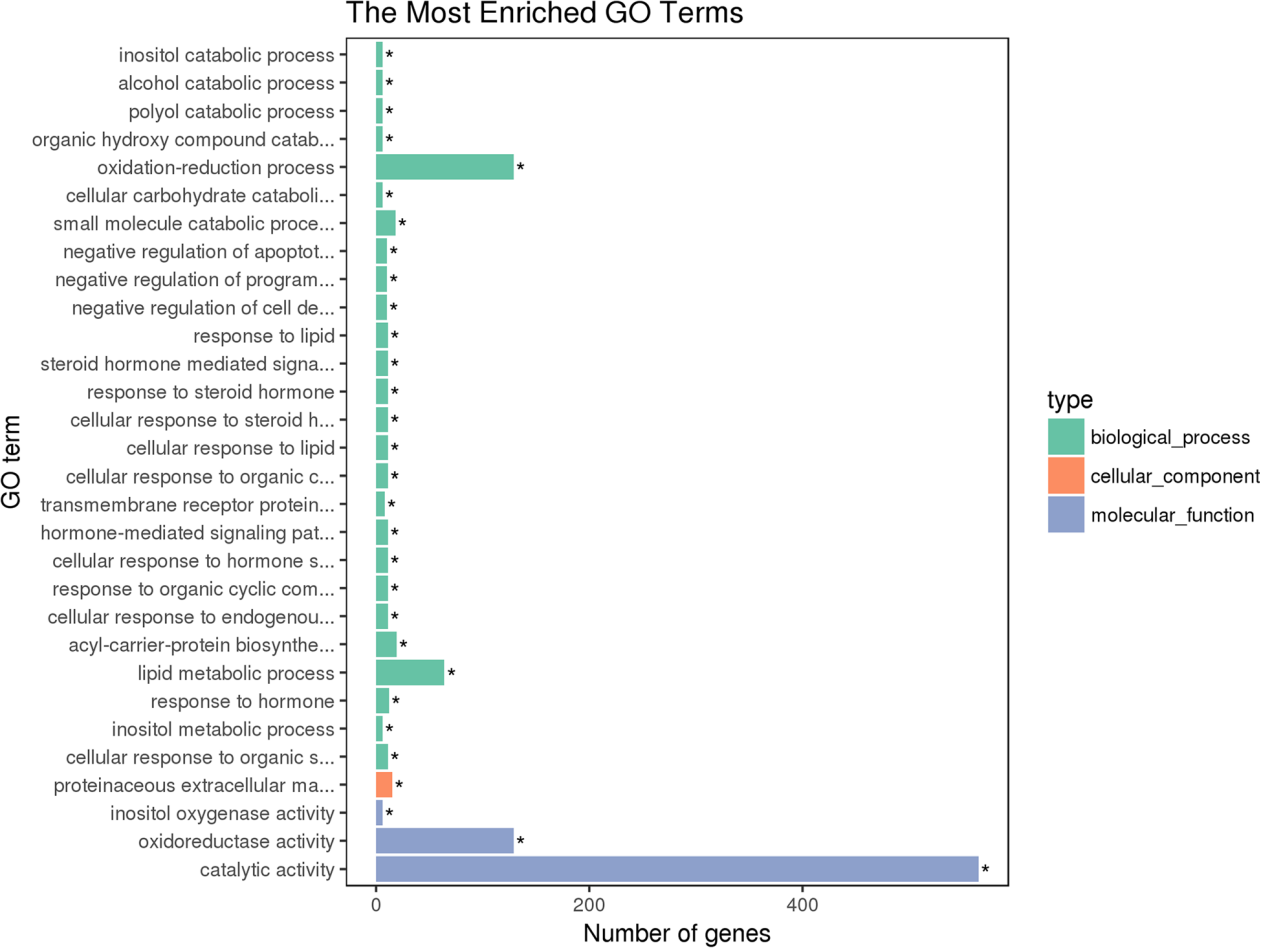
Gene Ontology (GO) classification of downregulated unigenes from the Cry1C-resistant strain of *C. suppressalis*

The downregulated unigenes were annotated to 1540, 331 and 603 GO terms for biological process, cellular component and molecular function, respectively. Among the top 30 GO terms (according to corrected *P*-value) 26 terms were for biological process, 1 for cellular component and 3 for molecular function (Fig. 6). With regard to the 26 biological process GO terms, inositol catabolic process was the most enriched category (GO: 0019310, corrected *P-*value = 1.35e^− 05^), and the oxidation-reduction process (GO: 0055114, corrected *P-*value = 7.24e^− 05^) was the largest, which included 129 downregulated unigenes. In terms of cellular components, the proteinaceous extracellular matrix was the most enriched term (GO: 0005578, corrected *P*-value = 0.017839), with 15 downregulated unigenes. Among the 3 molecular function GO terms, inositol oxygenase activity (GO: 0050113, corrected *P*-value = 1.35e^− 05^) was the most represented, and catalytic activity (GO: 0003824, corrected *P*-value = 0.0053335) was the largest, with 565 downregulated unigenes.

### Validation of differentially expressed genes by qRT-PCR

**Fig. 7:**
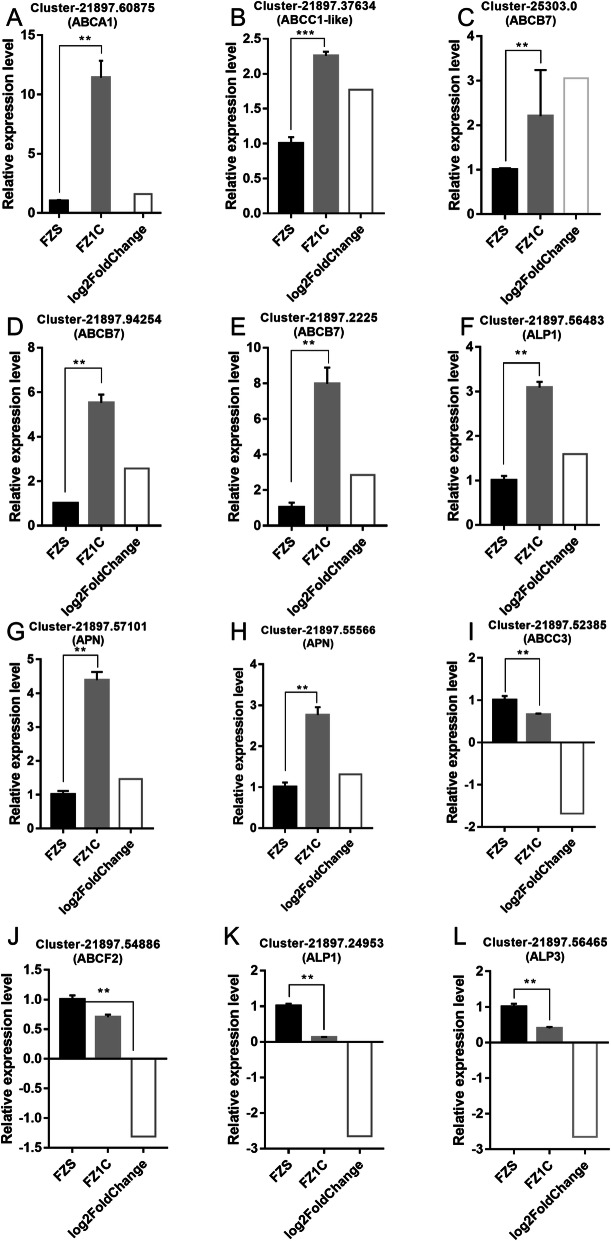
qRT-PCR analysis of twelve selected unigenes annotated to potential Cry toxin receptors to confirm the expression patterns indicated by sequencing. Quantitative real-time PCR analysis data of 12 selected genes are presented. Four technical replicates were performed for each of three biological replicates. The height of FZS and FZ1C boxes represent the mean average of sample-specific 2^-ΔΔCt^ values, and the log2FoldChange box represents the log2FoldChange value between FZS and FZ1C of specific unigenes. (A-L) Represent the relative expression level of Cluster-21,897.60875 (ABCA1), Cluster-21,897.37634 (ABCC1-like), Cluster-25,303.0 (ABCB7), Cluster-21,897.94254 (ABCB7), Cluster-21,897.2225 (ABCB7), Cluster-21,897.56483 (ALP1), Cluster-21,897.57101 (APN), Cluster-21,897.55566 (APN), Cluster-21,897.52385 (ABCC3), Cluster-21,897.54886 (ABCF2), Cluster-21,897.24953 (ALP1) and Cluster-21,897.56465 (ALP3), respectively. Values shown are means and standard errors

To validate the expression patterns of the unigenes, 12 differentially expressed unigenes (including Cluster-21,897.60875, 21,897.37634, 25,303.0, 21,897.94254, 21,897.2225, 21,897.56483, 21,897.57101, 21,897.55566, 21,897.52385, 21,897.54886, 21,897.24953 and 21,897.56465) were selected for qRT-PCR according to their fold change values in the expression profiles from the dataset. The elongation factor was set as the candidate reference gene for RT-qPCR normalization. The results showed that the expression patterns presented by the comparative transcriptome were consistent with the qRT-PCR results (Fig. 7). Overall, relative expression of the top 8 unigenes was significantly upregulated in the resistant FZ1C strain of *C. suppressalis*, whereas that of the last 4 unigenes was remarkably reduced in the resistant strain (Fig. 7).

## Discussion

The mode of Cry toxin action is a very complex process involving the toxin structure, many enzymes and receptors, among others, and a change in any step of the toxicology process inevitably leads to insect resistance. In this study, comparative transcriptome analysis was conducted between susceptible and resistant strains of *C. suppressalis*. A total of 139,206 unigenes were de novo assembled from 373 million clean reads in the *C. suppressalis* transcriptome. Among them, 5328 significantly DEGs were identified between *C. suppressalis* Cry1C-resistant and Cry1C-susceptible strains. Among these DEGs, unigenes encoding Bt Cry toxin receptors, aminopeptidase-P like protein, the ABC subfamily and alkaline phosphatase were downregulated and suggested to be associated with *C. suppressalis* Cry1C resistance. Additionally, the transcription level of unigenes encoding enzymes involved in hydrolysis, digestive, catalytic and detoxification processes were significant increased, which appeared to be related to Cry1C resistance in *C. suppressalis*. Presumably, multiple genes and different pathways are involved in Cry1C toxin resistance.

Among these DEGs, unigenes associated with the Cry toxin receptor were identified, such as aminopeptidase N, ABC transporter family members, alkaline phosphatase, cadherin, trypsin and V-type proton ATPase catalytic subunit A. Many previous studies on glycosylphophatidylinositol (GPI)-anchored APN1 from several insects have consistently demonstrated that APN1 is an important midgut receptor for Cry toxins and is associated with insect resistance [25, 43–46]. Our previous study found that knockdown of aminopeptidase-N isoform (APNs) results in decreased susceptibility of *C. suppressalis* larvae to Cry1A toxins, indicating that APNs are important functional receptors of Cry1A toxins in *C. suppressalis* [36]. Qiu et al. [37] demonstrated that downregulation of APNs by RNAi reduced the susceptibility of *C. suppressalis* larvae to Cry1C and Cry1Ab/Cry1Ac fusion proteins expressed by transgenic *Bt* rice, suggesting that APNs are involved in the mode of *Cry1Ab*/*Cry1Ac* and *Cry1C* action. In this study, five unigenes were annotated to APN1 (ABC69855.3), which included Cluster-21,897.55689, Cluster-21,897.55577, Cluster-21,897.55566, Cluster-21,897.49282 and Cluster-21,897.54390. One unigene annotated to APN2 (AGG36452.1) was Cluster-21,897.57790. Three unigenes were annotated to APN5 (AFU51581.1), which contained Cluster-21,897.54666, Cluster-21,897.56678 and Cluster-21,897.57101. Nine unigenes encoding APNs in the current study were all upregulated in the resistant strain (FZ1C) compared with the susceptible strain (FZS) of *C. suppressalis*. These results are consistent with those of previous studies [41, 47, 48]. Presumably, these upregulated APNs may compensate for the fitness costs of resistance [23]. In addition, we observed significant downregulation of two unigenes annotated as aminopeptidase P-like proteins in the resistant strain (FZ1C) of *C. suppressalis*; in particular, expression of one unigene was decreased 10.74-fold in the FZ1C strain compared with the FZS strain. Khajuria et al. [49] reported that an aminopeptidase P-like protein is possibly involved in Cry resistance in *O. nubilalis*. These results indicate that an aminopeptidase P-like protein is likely involved in the mode of Cry1C toxin action and accounts for *C. suppressalis* resistance to Cry1C.

Another important group of receptors involved in the mode of Cry toxin action is ABC transporters. Many studies have proven that the resistance of three lepidopteran species, *H. virescens*, *P. xylostella* and *Trichoplusia ni,* to *Cry1Ac* is linked to ABC transporters [27, 50]. ABC transporters, such as ABCG1, ABCC2, and ABCC3, are reported to be Cry toxin receptors, and their mutation or downregulation is proven to be related to insect resistance [25, 51, 52]. In *P. xylostella*, mutations in ABCC2 disrupt the binding of Cry1Ac to membrane vesicles and lead to Cry toxin resistance. Transcriptome analysis of *P. xylostella* showed that eight unigenes annotated as ABCC2 were detected in the Cry1Ac-resistant strain, with the majority being downregulated [41]. In this study, we identified 17 unigenes encoding ABC transporter family members, including ABCA1, ABCB1, ABCB7, ABCC1, ABCC3, ABCC4, ABCD2, ABCF2, ABCG1 and ABCG4, but no differentially expressed unigenes were annotated as ABCC2. Furthermore, six downregulated unigenes annotated as ABC transporters (Cluster-21,897.52385, Cluster-21,897.87849, Cluster-21,897.77904, Cluster-21,897.854, Cluster-21,897.54886 and Cluster-21,897.58674) were identified in the *C. suppressalis* resistant (FZ1C) strain and were considered to be associated with the resistance of *C. suppressalis* to Cry1C toxin. Nevertheless, further study should be conducted to reveal the exact function of ABC transporters in the resistant strain of *C. suppressalis*.

Previous studies have also demonstrated that ALP is an important receptor that interacts with Cry toxins [53–56]. In this study, we identified 10 unigenes matching ALP1, ALP3 and ALP-like that were significantly differentially expressed between Cry1C-resistant and Cry1C-susceptible strains of *C. suppressalis*. Among them, 7 unigenes (Cluster-21,897.109470, Cluster-21,897.58470, Cluster-21,897.75922, Cluster-21,897.56465, Cluster-21,897.58789, Cluster-21,897.40486 and Cluster-21,897.24953) were downregulated. We speculate that these unigenes with decreased transcript levels may account for *C. suppressalis* resistance to Cry1C toxin, in accordance with a previous study [42].

Three unigenes were annotated as cadherin (Cluster-21,897.53425, Cluster-21,897.66616 and Cluster-21,897.72833), but they were all upregulated in the resistant strain. This result is consistent with our previous study, which found that cadherin did not play a major role in the mode of Cry 1C action in *C. suppressalis* [35].

The conversion of the Cry protoxin to the cytotoxic form by proteases is an inevitable process for Cry toxicity. However, overexpression of midgut proteinases may enhance the possibility of degrading activated Cry toxin and cause a decrease in toxicity [40]. In this study, 12 unigenes annotated as trypsin or trypsin/chymotrypsinogen-like proteinase were overexpressed in the Cry1C-resistant strain compared with the susceptible strain. Similar results were also reported in *O. furnacalis* Cry1Ab-resistant strains [40] and Bt-resistant strains of mosquitoes [47]. Over-transcription of these proteinases in the resistant strain of *C. suppressalis* possibly reflects higher activity of midgut proteinases for degrading toxins. Additionally, some unigenes encoding serpin protease and serpin protease inhibitors were found to be associated with immune function for the regulation of haemolymph melanization. In addition, unigenes matching the detoxification enzymes cytochrome P450 (P450), carboxylesterase (CaE) and glutathione S-transferase (GST) were identified. In particular, P450 family members play an important role in degrading chemical insecticides. They have also been observed to respond to Cry toxin in lepidopterous insects [40, 57]. In the midgut transcriptome of *O. nubilalis* larvae [57], six unigenes encoding P450 were found to be downregulated and eight unigenes upregulated after exposure to Cry toxins. However, in *O. furnacalis* [40], eight unigenes matching P450 were all downregulated in a Cry toxin-resistant strain. Thus, the exact role of P450 in the mode of Cry toxin action is uncertain, as they are generally thought to be involved in the degradation of xenobiotics [58]. In the present study, a large proportion of unigenes (12/21) annotated as detoxification enzymes were upregulated in the Cry1C-resistant strain. These enzymes for detoxification may act as a general response to environmental stress (such as Cry toxin) or may help insects avoid the damage of Cry toxins and compensate for fitness costs [40, 57].

GO and KEGG analyses provided important clues to reveal the potential mechanisms involved in the development of resistance. In our study, a large number of unigenes showed significant enrichment in digestive enzymes, hydrolase enzymes, detoxification enzymes, and catalytic enzyme-related GO categories (Additional file 5). Previous studies reported that these enzymes are considered to be the most important factors in reducing fitness costs [17, 19, 41, 59]. These findings suggest that *C. suppressalis* resistance to Cry1C toxin may be associated with increased digestive activity, catalytic activity, detoxification activity and hydrolase activity in the larval midgut. Moreover, KEGG category analyses revealed significantly enrichment of a series of unigenes in xenobiotic stimulation and immunity-related KEGG pathways (Additional file 6). Similar findings were also found for *O. furnacalis* [40, 57] and *H. armigera* [42]. These results also indicated that Cry1C resistance may be associated with the immune response of *C. suppressalis*.

## Conclusions

In conclusion, this study is the first to report the mechanism of *C. suppressalis* resistance to Cry toxin based on genetic information from the sequenced transcriptome, revealing a large number of unigenes with greatly enriched sequence information for *C. suppressalis*. Based on our findings, several factors are related to Cry1C toxin resistance in this pest. The candidate receptors aminopeptidase-P-like protein, the ABC subfamily and alkaline phosphatase, which were downregulated, might be associated with Cry1C resistance in *C. suppressalis*. Additionally, changes in enzymes related to digestive activity, catalytic activity, detoxification activity, hydrolase activity and related genes may account for *C. suppressalis* resistance to Cry1C toxin. We also identified multiple pathways that might be involved in Cry1C resistance in *C. suppressalis*. Therefore, we speculate that the mechanism of *C. suppressalis* resistance to Cry1C toxin is a complicated process involving a series of genetic and metabolic factors. With these important genetic resources, further studies should be conducted to validate the gene functions associated with Bt resistance in *C. suppressalis* using RNA interference or genome-editing technology.

## Methods

### Insect rearing and selection of the resistant strain

The Fuzhou susceptible strain (FZS) of *C. suppressalis* was initially collected from paddy fields in Fuzhou (26.1°N, 119.3°E), Fujian Province, China, in 2012. The insect colony was continually reared on an artificial diet as described previously [1] without exposure to any Cry toxins. Based on the FZS strain, the Cry1C-resistant strain (FZ1C) was obtained from the fifth generations colony of the susceptible strain selected with an artificial diet with trypsin-activated Cry1C toxin (Meiyan (Beijing) Agricultural Technology Co., Ltd.). The Cry1C-selected strain (FZ1C) was initially exposed to a sublethal dose of the Cry1C diet throughout larval development. The toxin concentration was steadily increased in succeeding generations to achieve 40–60% mortality of the exposed larvae. After 41 generations of selection, the strain (FZ1C) developed 42.6-fold resistance [60]. In this study, the Cry1C-resistant strain (FZ1C) reared on an artificial diet containing 80 μg/mL activated Cry1C toxin, which had been selected for 57 generations with 120-fold resistance, was used to detect *C. suppressalis* Cry1C resistance-related genes. In parallel, the susceptible strain (FZS) reared in the absence of Cry1C toxin was used as the negative control.

All cultures were maintained under constant conditions (27 ± 1 °C, 70–80% RH, and a 16:8-h light/dark photoperiod).

### Dissection of the midgut and extraction of RNA

Before dissection, the FZS strain was reared on the diet without any Cry toxins or other chemical insecticides, and the FZ1C strain selected for 57 generations was reared on the artificial diet with 80 μg/mL activated Cry1C toxin. Larval midguts of both strains of *C. suppressalis* were dissected when the larvae reached the 4th instar. The respective midgut tissues were washed with 0.7% NaCl (w/v) to remove debris and haemolymph, and the samples were stored in RNA holding solution (TransGen Biotech Co. Ltd., Beijing, China). Thirty individual midguts from 4th-instar larvae of FZS and FZ1C were collected in an RNase-free 1.5 mL tube as one biological replicate and each strain contained six biological replicates. Three replicates were used to Illumina RNA-Seq and the gene expression profile analysis, the others were used for RT-qPCR analysis to validate the results of differentially expressed genes. TRIzol reagent was used to extract total RNA from each sample according to the manufacturer’s instructions, in which RNase-free DNase I was used to treat all samples to remove genomic DNA contamination. The RNA was suspended in 100 μL DEPC-treated water. After assessment of the quantity and purity of the total RNA, the RNA samples were delivered to Beijing Novogene Technology Company for RNA sequencing.

### RNA-Seq library preparation and Illumina sequencing

The RNA-Seq libraries were generated from a total amount of 1.5 μg RNA per biological replicate using NEBNext® Ultra™ RNA Library Prep Kit for Illumina® (NEB, USA) according to the manufacturer’s instructions, and index codes were added to identify the sequences of each sample. Briefly, mRNA was purified from total RNA using poly-T oligo-attached magnetic beads and fragmented by using divalent cations under elevated temperature. Synthesis of first-strand cDNA was performed using random hexamer primers and RNase H. Second-strand cDNA synthesis was subsequently performed using DNA polymerase I and RNase H. The remaining overhangs of the cDNA was converting into blunt ends by exonuclease/polymerase activities of DNA polymerase I, the 3′ ends of the DNA fragments were adenylated and NEBNext adaptors with hairpin loop structures were ligated. In order to obtain cDNA fragments of 250 ~ 300 bp in length, the cDNA library fragments were purified with the AMPure XP system (Beckman Coulter, Beverly, USA). Subsequently, the cDNA fragments were treated with adding 3 μL USER Enzyme (NEB, USA) and incubated at 37 °C for 15 min followed by 5 min at 95 °C before Polymerase Chain Reaction (PCR) reaction. The cDNA fragments were amplificated by PCR using Phusion High-Fidelity DNA polymerase, universal PCR primers and index Primer. After purified by AMPure XP system, the library quality was evaluated using Agilent Bioanalyzer 2100 system.

The cBot Cluster Generation System was applied to cluster the index-coded samples using a TruSeq PE Cluster Kit v3-cBot-HS (Illumina) according to the manufacturer’s protocols. After cluster generation, the library preparations were sequenced using the Illumina HiSeq 4000 platform to generate 150 bp paired-end raw reads. The clean reads were obtained by removing reads containing adapters, reads containing poly-N and low-quality reads from the raw reads by first processing through in-house Perl scripts. At the same time, the Q20, Q30, GC content and sequence duplication level of the clean reads were calculated in this step. All of the downstream analyses were conducted with clean reads of high quality.

### Annotation of assembled unigenes

Transcriptome assembly was accomplished based on the reads using Trinity [61] with min_kmer_cov set to 2 by default and the other parameters set to default. The assembled sequences were analysed in Nr database (NCBI non-redundant protein sequences) (https://www.ncbi.nlm.nih.gov/genbank/ and https://www.ncbi.nlm.nih.gov/protein/) and Nt database (NCBI non-redundant nucleotide sequences) (https://www.ncbi.nlm.nih.gov/nucleotide/) to identify the potential descriptions. The conserved domain was analysed by searching the Pfam database (Protein family) (https://pfam.sanger.ac.uk/). GO enrichment was conducted based on the GO database (Gene Ontology) (http://www.geneontology.org/). The KOG and KEGG annotation were performed against the KOG database (https://www.ncbi.nlm.nih.gov/COG/) and KEGG database (http://www.genome.jp/kegg/).

### Identification of differentially expressed genes (DEGs)

Gene expression levels were estimated by RSEM [62]. The DESeq R package (1.10.1) was applied to analyse differentially expressed genes (DEGs) between the susceptible and resistant strains basing on the negative binomial distribution model. The resulting *P* values were adjusted using Benjamini and Hochberg’s approach for controlling the false discovery rate. Genes with an threshold of |log2(fold- change)| and adjusted *P* value (*P* < 0.05) found by DESeq were considered significantly differentially expressed.

### Validation of differentially expressed genes by real-time quantitative PCR

Transcriptome results were verified using quantitative real-time PCR (qRT-PCR). Total RNA was extracted from each sample using TRIzol reagent according to the product instructions, with RNase-free DNase I to digest all samples to avoid genomic DNA contamination. The RNA was suspended in 100 μL DEPC-treated water. First-strand cDNA was synthesized by a Fast Quant RT Kit (With gDNase, KR106) (TIANGEN Biotech Co. Ltd., Beijing, China). Twelve genes (Additional file 7) identified as differentially expressed were selected based on their fold changes in the expression profile. Primer pairs (Additional file 8) were designed using Primer3plus (http://www.bioinformatics.nl/cgi-bin/primer3plus/primer3plus.cgi). qPCR was performed using the ABI 7500 system (ABI, USA) with a reaction volume of 20 μL containing 1 μL of 1:5 diluted cDNA in RNase-Free ddH_2_O, 10 μL 2 × SuperReal PreMix Plus (SYBR Green, FP205, TIANGEN Biotech Co. Ltd., Beijing, China), 0.6 μL of 10 μM each primer, 0.4 μL of 50× ROX reference dye, and 7.4 μL of RNase-Free ddH_2_O. The qPCR conditions were 95 °C for 15 min, followed by 40 cycles of 95 °C for 10 s for denaturation and 60 °C for 32 s for annealing and extension. The melting curve was produced at 95 °C for 15 s, 60 °C for 1 min, 95 °C for 15 s, and 60 °C for 15 s. Elongation factor 1 (EF-1) was used as an internal reference gene. Each of the three biological replicates was measured with four technical replicates, and the expression levels of candidate genes were normalized with the EF-1 gene.

## Supplementary information

**Additional file 1: Figure S1.** Annotation of assembled unigenes of Cry1C-resistant and -susceptible strains of *C. suppressalis* in different databases.

**Additional file 2: Figure S2.** Species classification from homology analysis of assembled unigenes from Cry1C-resistant and -susceptible strains of *C. suppressalis*.

**Additional file 3: Figure S3.** Hierarchical cluster analysis of C. suppressalis midgut samples from Cry1C-resistant (FZ1C) and Cry1C-susceptible (FZS) strains. FZ1Ca_1, FZ1Ca_2, FZ1Ca_3: three sample replications from the FZ1C strain; FZS_1, FZS_2, FZS_3: three sample replications from the FZS strain.

**Additional file 4: Figure S4.** Heatmap of differentially expressed unigenes among candidate resistance genes from Cry1C-resistant and -susceptible strains of *C. suppressalis*. (A) ABC transporter. (B) Alkaline phosphatases. (C) Aminopeptidase-N and aminopeptidase P-like proteins. (D) Cadherins. (E) Heat shock proteins. (F) Serine protease inhibitors. (G) Serine proteases. (H) Trypsins. (I) V-ATPase.

**Additional file 5:.** GO enrichment of DEGs.

**Additional file 6:.** KEGG enrichment of DEGs.

**Additional file 7:.** The sequences of twelve unigenes selected for qRT-PCR.

**Additional file 8:.** Primer sequences for qRT-PCR of twelve selected unigenes annotated as potential Cry toxin receptors.

## Data Availability

The raw reads of the two transcriptomes in this study have been deposited in the NCBI SRA database under accession numbers SRR12170219 (midgut transcriptome of susceptible strain of *Chilo suppressalis*) and SRR12170218 (midgut transcriptome of Cry1C resistant strain of *Chilo suppressalis*).

## Abbreviations

*Bt*: *Bacillus thuringiensis*
DEG: Differentially expressed unigenes (DEGs)
ABC: ATP-binding cassette
IRM: Insect resistance management
GPI: Glycosylphosphatidylinositol
APN: Aminopeptidase N
ALP: Alkaline phosphatase
RNA-Seq: RNA-sequencing
FZ1C: Fuzhou Cry1C-resistant
FZS: Fuzhou Cry-susceptible
APP-like: Aminopeptidase P-like protein
CaE: Carboxylesterase
GST: Glutathione S-transferase
P450: Cytochrome P450 monooxygenase
