# Supplementary figures and images for: Differences in midgut transcriptomes between resistant and susceptible strains of Chilo suppressalis to Cry1C toxin
Source: BMC Genomics. 2020 Sep 14;21:634. doi: 10.1186/s12864-020-07051-6 (PMC7490912; doi:10.1186/s12864-020-07051-6)

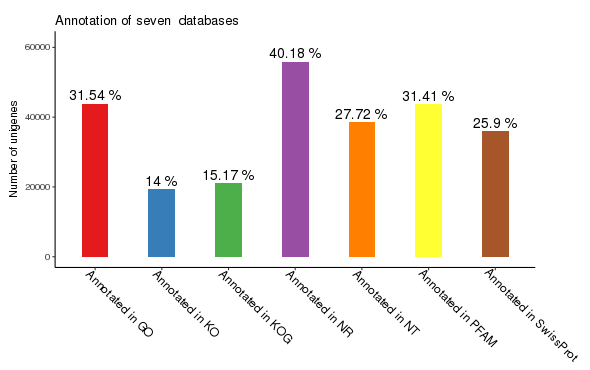

Supplement: Supplementary file 1 — Additional file 1: Figure S1. Annotation of assembled unigenes of Cry1C-resistant and -susceptible strains of C. suppressalis in different databases. [file 12864_2020_7051_MOESM1_ESM.png]

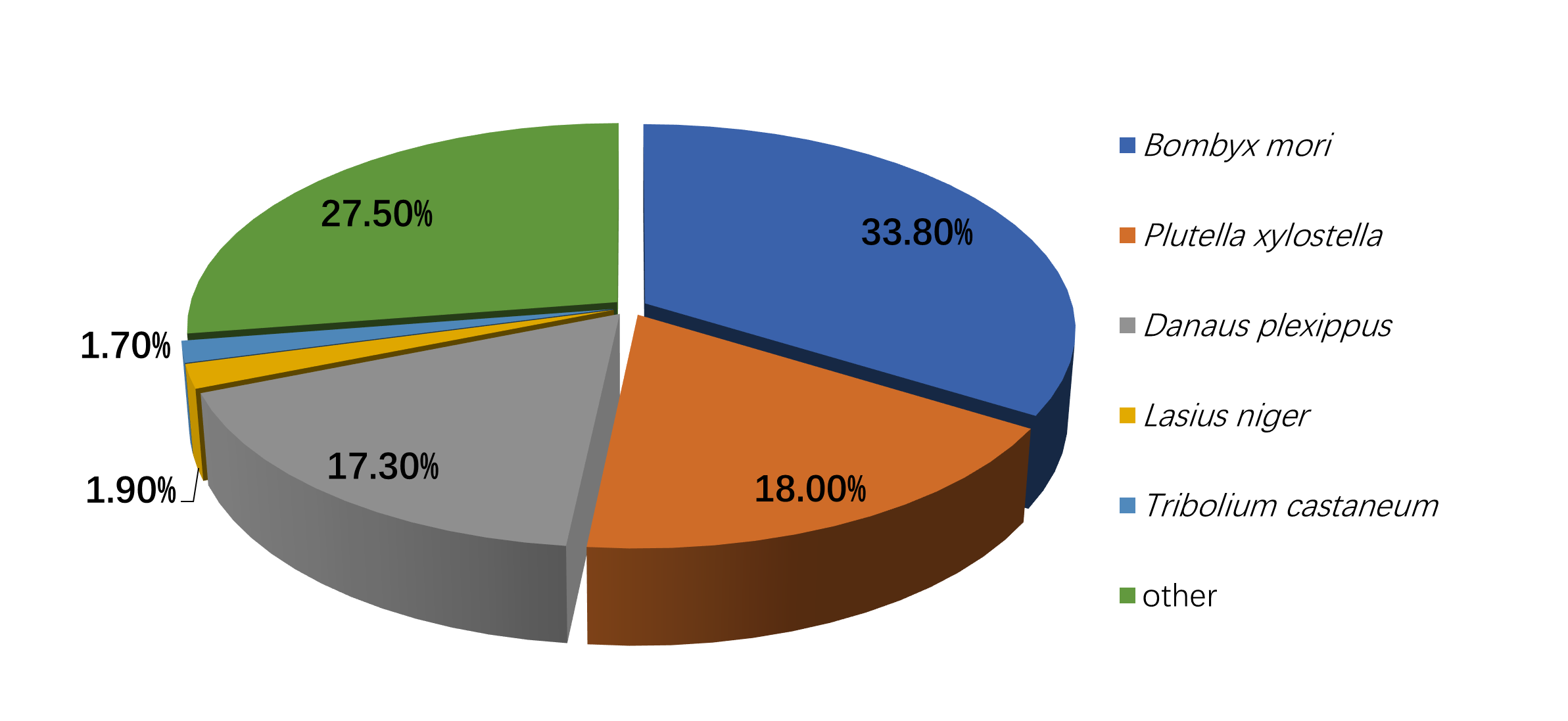

Supplement: Supplementary file 2 — Additional file 2: Figure S2. Species classification from homology analysis of assembled unigenes from Cry1C-resistant and -susceptible strains of C. suppressalis. [file 12864_2020_7051_MOESM2_ESM.tif]

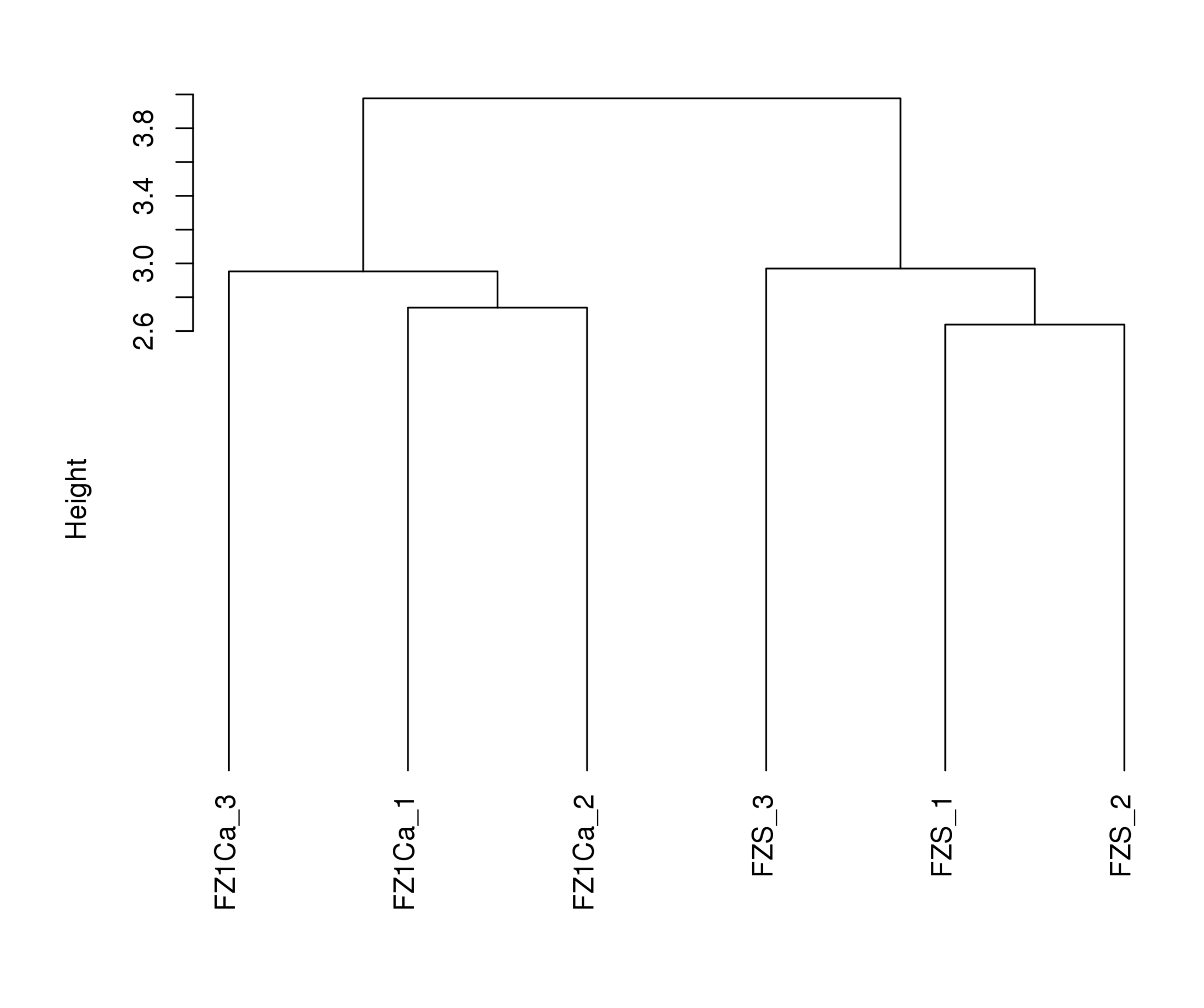

Supplement: Supplementary file 3 — Additional file 3: Figure S3. Hierarchical cluster analysis of C. suppressalis midgut samples from Cry1C-resistant (FZ1C) and Cry1C-susceptible (FZS) strains. FZ1Ca_1, FZ1Ca_2, FZ1Ca_3: three sample replications from the FZ1C strain; FZS_1, FZS_2, FZS_3: three sample replications from the FZS strain. [file 12864_2020_7051_MOESM3_ESM.tif]

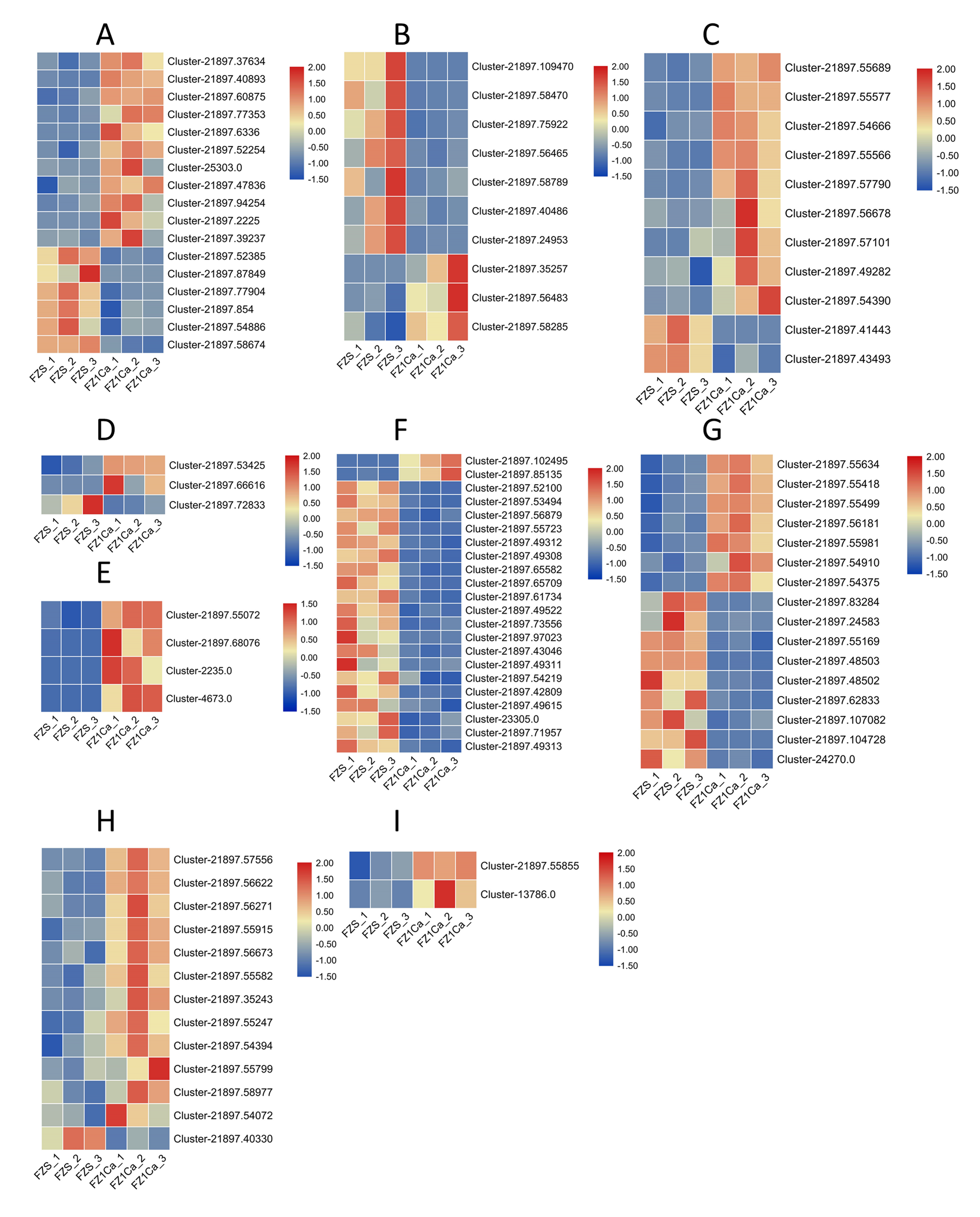

Supplement: Supplementary file 4 — Additional file 4: Figure S4. Heatmap of differentially expressed unigenes among candidate resistance genes from Cry1C-resistant and -susceptible strains of C. suppressalis. (A) ABC transporter. (B) Alkaline phosphatases. (C) Aminopeptidase-N and aminopeptidase P-like proteins. (D) Cadherins. (E) Heat shock proteins. (F) Serine protease inhibitors. (G) Serine proteases. (H) Trypsins. (I) V-ATPase. [file 12864_2020_7051_MOESM4_ESM.tif]
